# A Hybrid NF-FO-RO Process for the Supply of Irrigation Water from Treated Wastewater: Simulation Study

**DOI:** 10.3390/membranes11030191

**Published:** 2021-03-10

**Authors:** MhdAmmar Hafiz, Radwan Alfahel, Alaa H. Hawari, Mohammad K. Hassan, Ali Altaee

**Affiliations:** 1 Department of Civil and Architectural Engineering, Qatar University, Doha 2713, Qatar; mh1201889@qu.edu.qa (M.H.); ra1404482@qu.edu.qa (R.A.); 2 Center for Advanced Materials, Qatar University, Doha 2713, Qatar; mohamed.hassan@qu.edu.qa; 3 School of Civil and Environmental Engineering, University of Technology in Sydney, 15 Broadway, Ultimo, NSW 2007, Australia; Ali.Altaee@uts.edu.au

**Keywords:** forward osmosis, reverse osmosis, nanofiltration, municipal wastewater, irrigation

## Abstract

Municipal treated wastewater could be considered as a water source for food crop irrigation purposes. Enhancing the quality of treated wastewater to meet irrigation standards has become a necessary practice. Nanofiltration (NF) was used in the first stage to produce permeate at relatively low energy consumption. In the second stage, two membrane combinations were tested for additional water extraction from the brine generated by the NF process. The simulation results showed that using a hybrid forward osmosis (FO)–reverse osmosis (RO) system is more efficient than using the RO process alone for the further extraction of water from the brine generated by the NF process. The total specific energy consumption can be reduced by 27% after using FO as an intermediate process between NF and RO. In addition, the final permeate water quality produced using the hybrid FO-RO system was within the allowable standards for food crops irrigation.

## 1. Introduction

Treated sewage effluent (TSE) could be considered as a valuable source of water that might be used for the irrigation of food crops, especially in arid and semi-arid regions. However, the direct use of TSE could damage the soil because of the excessive salts, pathogens, colloids, phosphor, and nitrate content [1,2]. Enhancing the quality of TSE to meet irrigation standards has become a common practice. This must be done with minimal capital and operational costs. Membrane technologies have attracted a lot of attention in desalination and wastewater treatment due to their low energy requirement [3], small footprint [4], low operational cost [5], and high removal efficiency of various pollutants [6,7,8]. The most widely used membrane technologies are microfiltration (MF), ultrafiltration (UF), nanofiltration (NF), and reverse osmosis (RO). These processes have been used at an industrial scale for desalination and wastewater treatment. Forward osmosis (FO) is a promising membrane technology that depends on the osmotic pressure gradient between the low concentrated feed solution and the highly concentrated draw solution [6]. FO has low energy consumption and membrane fouling when compared to other membrane processes [9]. Forward osmosis has been used for various wastewater treatment applications, including fertigation [10,11,12,13], industrial water and wastewater treatment [14,15,16], and a pretreatment process for desalination using reverse osmosis [17,18,19].

The water recovery rate is used to measure the water production rate in any membrane process. The recovery rate is defined as the percentage of permeate separated from the feed water [20]. The recovery rate depends on the feed water quality and the performance of the membrane process [21]. It is always desirable to achieve a high recovery rate, but exceeding 75% in NF and RO could be difficult, especially when treating municipal wastewater due to membrane fouling and high energy consumption [22]. Therefore, the hybridization of membrane processes could increase the recovery rate, enhance the permeate water quality and reduce membrane fouling. Shanmuganathan et al. compared the performance of NF and RO for enhancing the quality of microfiltered treated sewage effluent [23]. The results showed that using RO or NF alone could not produce permeate which meets the irrigation water standards. However, irrigation suitable permeate was produced using an NF-RO hybrid system. It was found that blending NF permeate and RO permeate after NF pretreatment is a cost-effective option as the RO process is more expensive than NF, and only 50% of the NF permeate was treated by RO. Touati et al. designed a UF-NF-RO hybrid process to produce isotonic solutions and drinking water from coastal well water [24]. The brine produced by NF was treated using RO to be used as drinking water. The maximum recovery rate of the hybrid process was 75%, with a specific energy consumption of 8.5 kWh/m^3^. Oron et al. evaluated the performance of a pilot-scale hybrid UF–RO process to produce water suitable for irrigation from secondary treated municipal wastewater [25]. The maximum recovery rate of the hybrid process was almost 55%, and the operating cost was between 0.16 and 0.24 US$/m^3^ water. In our previous study, a hybrid FO–RO process was used to produce irrigation water from TSE [26]. The feed solution and draw solution for the FO was TSE and an engineered fertilizing solution, respectively. The diluted draw solution produced by the FO process was regenerated using the RO process. The recovery rate of the FO process was between 3% and 4%, and the specific power consumption was between 2.18 and 2.58 kWh/m^3^. Al-Amoudi et al. evaluated the performance of the dual stage NF process for seawater desalination [27]. The maximum recovery rate was 22%, with an energy consumption of 4.2 kWh/m^3^. Chon et al. used a hybrid technology of membrane bioreactor and nanofiltration to produce irrigation water from municipal wastewater [28]. It was found that the physicochemical properties and membrane molecular weight cut-off were the most critical aspects in the removal of nutrients from the water.

In this paper, a hybrid NF-FO-RO process was simulated to produce irrigation water from tertiary treated municipal wastewater. The proposed hybrid system is designed to achieve a recovery rate of 90%. The hybrid process includes two stages. In the first stage, TSE was used as the feed solution to the NF process. In the second stage, more freshwater was reclaimed from the concentrated brine generated by NF using two alternatives. In the first alternative, the concentrated brine was pumped to a RO process, and the permeate water was mixed with the permeate generated in the first stage to produce the final product water for irrigation purposes. In the second alternative, the concentrated brine was pumped to a FO process as the feed solution, and the draw solution was a synthetic saline solution with salinity ranging between 0.25 and 0.5 M. The diluted draw solution was pumped to a RO process for regeneration, the RO permeate was mixed with NF permeate generated in the first stage which is the final product water to be used for irrigation purposes. The simulated product water quality was compared to the irrigation water standards provided by the Food and Agriculture Organization (FAO) [29]. The water application value engine (WAVE) was used to predict the performance of NF and RO processes. A predeveloped software was used to predict the performance of the FO process [30].

## 2. Materials and Methods

### 2.1. Methodology

**Figure 1:**
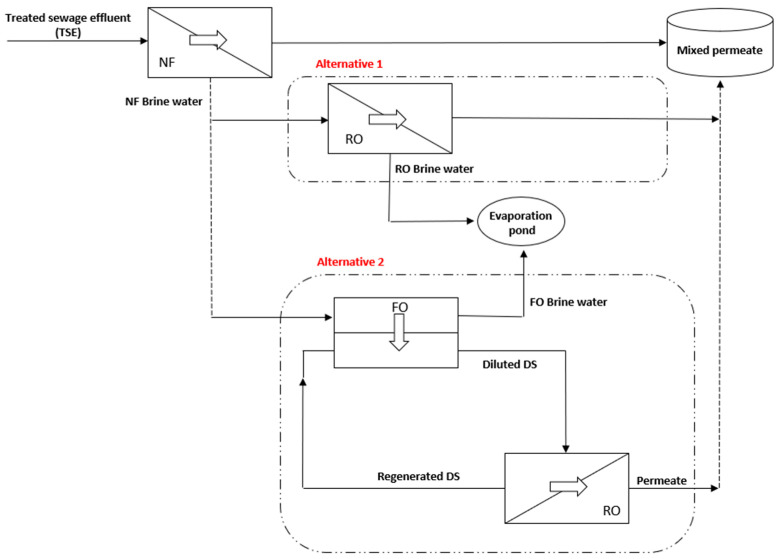
A schematic sketch showing the hybrid process used in this study.

The hybrid NF-FO-RO is a multistage membrane process suggested to produce irrigation water using TSE. In stage 1, TSE was used as a feed solution in the NF process. Nanofiltration membrane (Filmtec NF 90–400) produced by Dupont (Wilmington, DE, USA) was used in the first stage. NF90 has high productivity performance while removing a high percentage of divalent ions, nitrate, iron, and organic compounds, such as pesticides and herbicides [31,32]. The feed water flow rate was 200,000 m^3^/d. The low net driving pressure of the NF90 membrane allows the removal of these compounds at low operating pressures. Therefore, the NF permeate was expected to have low or moderate salinity. The number of NF vessels was 490, with 8 elements per vessel. In stage 2, the brine collected from NF was treated further to increase the overall recovery rate of the process and reduce the volume of the discharged brine. In this stage, the performance of two membrane combinations was compared, alternative 1: RO and alternative 2: FO-RO hybrid process. In alternative 1, the concentrated brine collected from NF was pumped to an RO process for additional water recovery using brackish water reverse osmosis membrane (BW30HRLE-440) manufactured by Dupont (Wilmington, DE, USA). Then the permeate water from the RO process was mixed with NF permeate obtained in the previous stage to form the final product. The brine water produced by the RO process was recycled back to the sewage treatment plant. The number of BWRO vessels was 313, with 8 elements per vessel. In alternative 2, the concentrated brine collected from NF was pumped to the FO process as a feed solution for additional water recovery. TFC FO membrane, FTSH2O (San Leandro, CA, USA), was used in this study. The number of FO vessels was 403 vessels, with 8 elements per vessel. The draw solution was a synthetic saline solution with salinity ranging between 0.25 and 0.5M. The FO process depends on the osmotic pressure gradient between the feed solution and draw solution. Using FO as an intermediate process has several advantages, such as low power consumption, low membrane fouling propensity, and high-water recovery. The recovery rate of the FO process was adjusted using the draw solution concentration. The diluted draw solution was pumped to the RO process for regeneration of the draw solution using seawater RO membrane (SW30HR–380) manufactured by Dupont (Wilmington, DE, USA). Permeate collected from the RO was mixed with NF permeate obtained in the previous stage to form the final product. The number of SWRO vessels was 167, with 8 elements per vessel. Figure 1 summarizes the two studied alternatives.

The water application value engine (WAVE) (Version 1.81) developed by Dupont (Wilmington, DE, USA) was used to predict the performance of NF and RO processes. A pre-developed software was used to predict the performance of the FO process [30]. The results obtained from WAVE were manually entered into the predeveloped FO simulation software. In the FO software, the following equations were used:

The osmotic pressure of the solution was calculated using Van’t Hoff equation (Equation (1)):

(1) π=iCRT

where π is the osmotic pressure (bar), *i* is the Van’t Hoff factor, *C* is the molar concentration of the solution (M), *R* is the ideal gas constant (0.08206 L atm mol^−1^ K^−1^), *T* is the temperature (303 K).

The water flux in the FO process was calculated using Equation (2):

(2) $$J_{w,FO}=A_{w}\;\left(\pi_{DS}-\pi_{FS}\right)$$

where, $J_{w,FO}$ is the water flux (LMH), *π_DS_* is the osmotic pressure of the draw solution (bar), *π_FS_* is the osmotic pressure of the feed solution (bar), and *A_w_* is the membrane permeability (0.792 L m^−2^ h^−1^ bar^−1^) [33,34]. The permeate flow rate was calculated using Equation (3).

(3) $$Q_{P}=J_{w}A_{m}$$

where, $Q_{p}$ is the permeate flow rate (m^3^/h), and *A_m_* is the area of the membrane (m^2^). The recovery rate *(Re%)* is the ratio of permeate flow rate to the feed flow rate as shown in Equation (4):

(4) $$Re\%=\;\frac{Q_{p}}{Q_{f}}\times 100$$

The salt permeability coefficient (*B*) was calculated using Equation (5):

(5) $$B=\;\frac{\left(1-R_{j}\right)}{R_{j}}\;J_{w}$$

where *R_j_* is the membrane rejection rate, the rejection rate for the monovalent ions was 98%, and the rejection rate for divalent ions was 99.5%. The concentration of permeate (*C_P_*) is the ratio of salt flux to water flux in the membrane as shown in Equation (6):

(6) $$C_{P}=\frac{J_{s}}{J_{w}}$$

According to Altaee et al. [35], the membrane salt permeability *(J_s_)* was calculated using Equation (7):

(7) $$J_{s}=B\;\left(C_{FS}-C_{p}\right)$$

Substituting Equation (7) into Equation (8) and rearrange to calculate the concentration of permeate as following:

(8) $$C_{P}=\frac{B\;C_{FS}}{J_{w}+B}$$

where *C_FS_* is the concentration of the feed solution in the FO process, it should be noted here that salt diffusion from the feed to the draw solution side of the membrane will affect the concentration of the draw solution. Therefore, the final concentration of the draw solution *(C_DS,in_)* is estimated using Equation (9):

(9) $$C_{DS,in}=C_{DS}+C_{P}\;\frac{Q_{P}}{Q_{DS,in}}$$

where *Q_DS,in_* is the draw solution flow rate (m^3^/h), and *C_DS_* is the initial draw solution concentration without the effect of salt diffusion. The concentration of the draw solution after the dilution using the FO process *(C_DS,out_)* was calculated using Equation (10):

(10) $$C_{DS,out}=\frac{C_{DS,in}}{1+\frac{Q_{P}}{Q_{DS,in}}}$$

Equations (1)–(10) can be used to calculate the flow and concentration of draw solution and feed solution in the FO process. The diffusion of salts from the draw solution to the feed solution is known as reverse solute flux. According to Philip et al. [36], reverse solute flux *(RSF)* was calculated using Equation (11).

(11) $$RSF=\frac{J_{w}\;C_{DS}}{1-\left(1+\frac{J_{w}}{B}\right)\exp\left(\;\frac{J_{w}\;S}{D}\right)}$$

where *S* is the membrane structural parameter (215 µm) and *D* is the draw solute diffusion coefficient (1.5 × 10^5^ m^2^/s) [33,37]. The energy consumption of the FO process (*E_s,FO_*) (kWh/m^3^) was calculated using Equation (12):

(12) $$E_{s,FO}=\left(\frac{\left(P_{DS}\right)\left(Q_{DS}\right)+\left(P_{FS}\right)\left(Q_{FS}\right)}{36\times n\times Q_{p}}\right)$$

where $P_{DS}$ and $P_{FS}$ are the draw solution and feed solution pressure (bar), respectively. $Q_{DS}$ and $Q_{FS}$ are the draw solution and feed solution flow rates (m^3^/h), respectively. *n* is the pump efficiency and assumed as 0.8.

### 2.2. Materials and Setup

In this study, ultra-filtered tertiary treated sewage effluent (TSE) was used as a feed solution to the hybrid process. A sample was collected from a wastewater treatment plant located in Doha, Qatar, to measure the water quality. The wastewater treatment plant consists of preliminary, secondary, and tertiary treatment processes. The tertiary treatment process consists of a multimedia filter followed by ultrafiltration and UV disinfection. The characteristics of the collected treated sewage effluent are summarized in Table 1. The use of TSE as irrigation water for food crops was unsuitable due to the excessive Total dissolved solids (TDS) and electrical conductivity (EC). The conductivity of the collected samples was measured using an OAKTON PCD650 multi-meter. The turbidity was measured using a turbidity meter (Hach 2100p).

## 3. Results and Discussion

### 3.1. Nanofiltration (NF) Process (Stage1)

**Figure 2:**
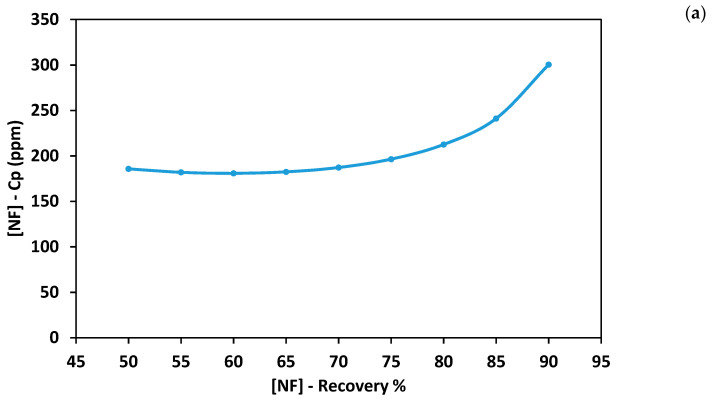

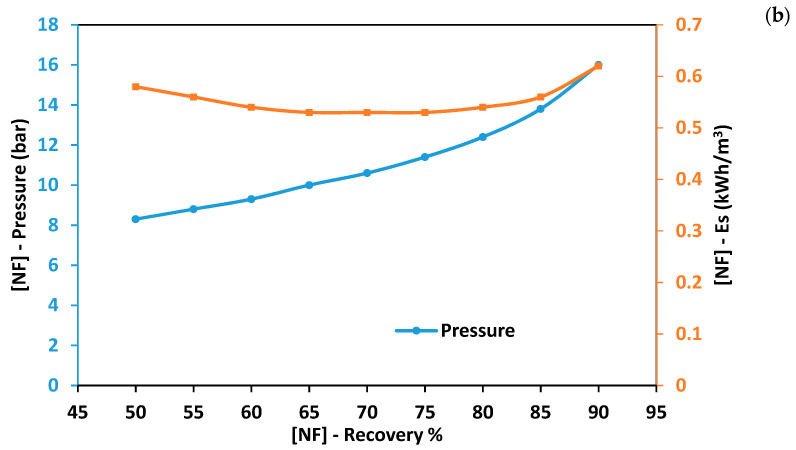
Performance of the nanofiltration (NF) process at different operating pressure. (**a**) concentration of permeate solution; (**b**) applied pressure and specific power consumption.

Nanofiltration was used in the first stage of the hybrid process. Treated wastewater was treated using NF. It can be seen from Figure 2a, the concentration of permeate (*C_P_*) was almost constant for a recovery rate between 50% and 75% and increased rapidly as the recovery rate increased above 80%. At a recovery rate between 50% and 70%, *C_P_* was almost 184 ppm, which is slightly higher than the TDS limit recommended by Food and Agriculture Organization. As the recovery rate increased to 75% and 80%, *C_P_* increased to 197 and 213 ppm, respectively. The maximum *C_P_* was 301 ppm obtained at a recovery rate of 90%; this is almost double the limit recommended by FAO. Overall, the concentration of permeate solution exceeded the TDS allowable limit, especially at high recovery rates. This is due to the fact that NF90 membranes have a loose structure with low rejection abilities for monovalent ions and a molecular weight cut-off (200–400) Da [41,42,43,44]. Figure 2b shows the relationship between the recovery rate and the applied pressure. The required applied pressure increased as the recovery rate increased. The minimum applied pressure was 8.3 bar, obtained at a recovery rate of 50%. The maximum applied pressure was 16 bar obtained at a recovery rate of 90%. NF feed pressure increased while increasing the recovery rate due to the accumulation of salts on the membrane surface, which increased the effect of concentration polarization [43]. It can be seen from Figure 2b, the specific power consumption (*E_s_*) decreased as the recovery rate increased between 50% and 80%. However, *E_s_* increased again at high recovery rates. The energy consumption was 0.58 kWh/m^3^ at a recovery rate of 50% and slightly decreased to 0.5 kWh/m^3^ at a recovery rate of 60%. The maximum energy consumption was 0.62 kWh/m^3^ obtained at a recovery rate of 90%. According to Equation (6), the specific power consumption should decrease as the recovery rate increase. In the current simulation experiments, the energy consumption increased as the recovery rate exceeded 80%. This can be attributed to the concentration polarization phenomena stated earlier that resulted in higher demand for applied pressure. The concentrated brine generated by NF shall be treated further to extract more fresh water. Two alternatives are proposed for the further treatment of the concentrated brine.

### 3.2. Treatment of Concentrated Brine Generated by the NF Process (Stage2)

#### 3.2.1. Reverse Osmosis (RO) Process (Stage 2: Alternative 1)

**Figure 3:**
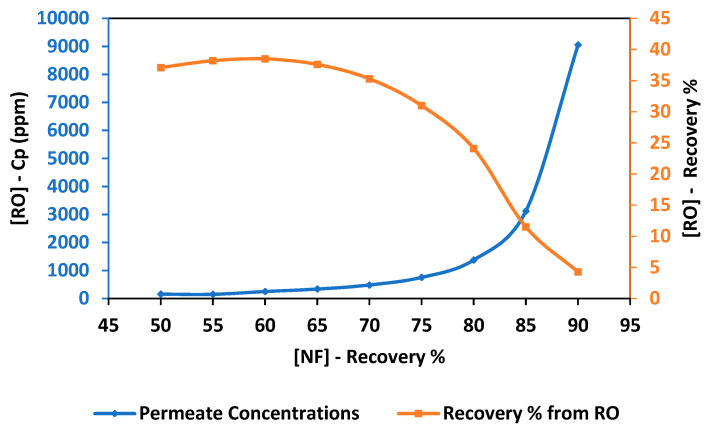
Permeate concentration and the recovery rate of reverse osmosis (RO) process at different concentrations of feed solution.

**Figure 4:**
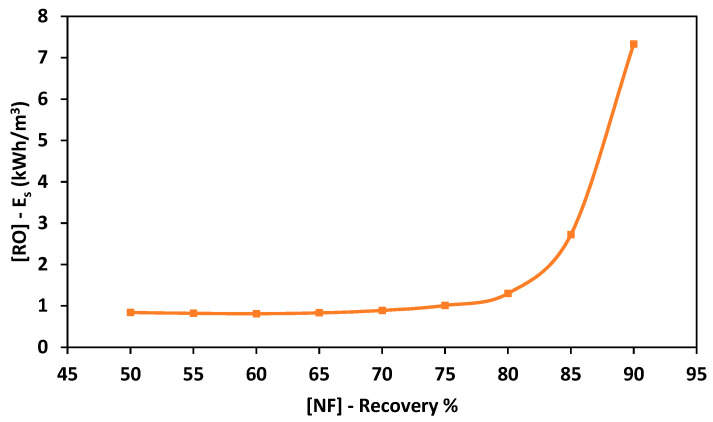
Energy consumption of the RO process using different concentrations of feed solution.

Reverse osmosis is proposed for the extraction of extra pure water from the concentrated brine reject generated by NF. As observed in Figure 3, the RO permeate concentration increased as the NF recovery rate increased. When using the concentrated brine obtained from NF at a recovery rate of 50% as a feed solution into the RO process, the concentration of RO permeate was 156 ppm. As the NF recovery rate increased to 60%, the concentration of RO permeate increased to 249.7 ppm. The concentration of RO permeate increased to 482 ppm as the NF recovery rate increased to 70%. At a recovery rate of 90%, the concentration of RO permeate increased significantly to 9057 ppm. The concentration of RO permeate depends on the membrane properties and salinity of the feed solution. Therefore, the concentration of the RO permeate was high when the highly concentrated brine solution was used as the feed solution. The recovery rate of RO was almost constant when using a feed solution with low salinity and decreased significantly when using highly concentrated brine as the feed solution. The recovery rate of RO was almost 37.8% when using the brine of NF obtained at a recovery rate between 50% and 65%. When using the concentrated brine obtained from the NF process at a recovery rate of 80%, the recovery rate of RO dropped to 24.7%. The minimum recovery rate of RO was 4% obtained using an NF recovery rate of 90%. This can be attributed to the excessive concentration polarization due to the high salinity of the used feed solution [45]. Figure 4 shows the relationship between the recovery rate from NF and the energy consumption of the RO process. The energy consumption was constant at 0.85 kWh/m^3^ when using an NF recovery rate between 50% and 70%. As the recovery rate of the NF process increased to 80%, the energy consumption of RO increased to 1.3 kWh/m^3^. At a recovery rate of 90%, the energy consumption of RO increased significantly by 7 times to a value of 7.33 kWh/m^3^. The energy consumption increased as the recovery rate of the NF process increased due to the higher applied pressure requirements [29].

**Figure 5:**
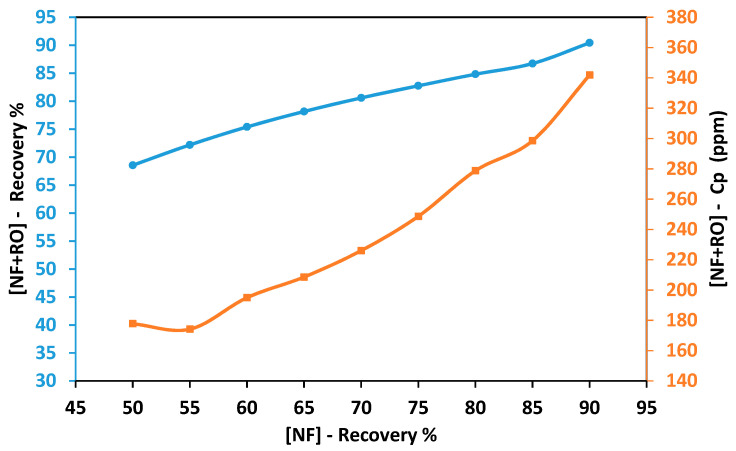
Total recovery rate and concentration of the mixed permeate solution obtained using hybrid Nanofiltration-Reverse osmosis (NF-RO) process at different feed solution concentrations.

**Figure 6:**
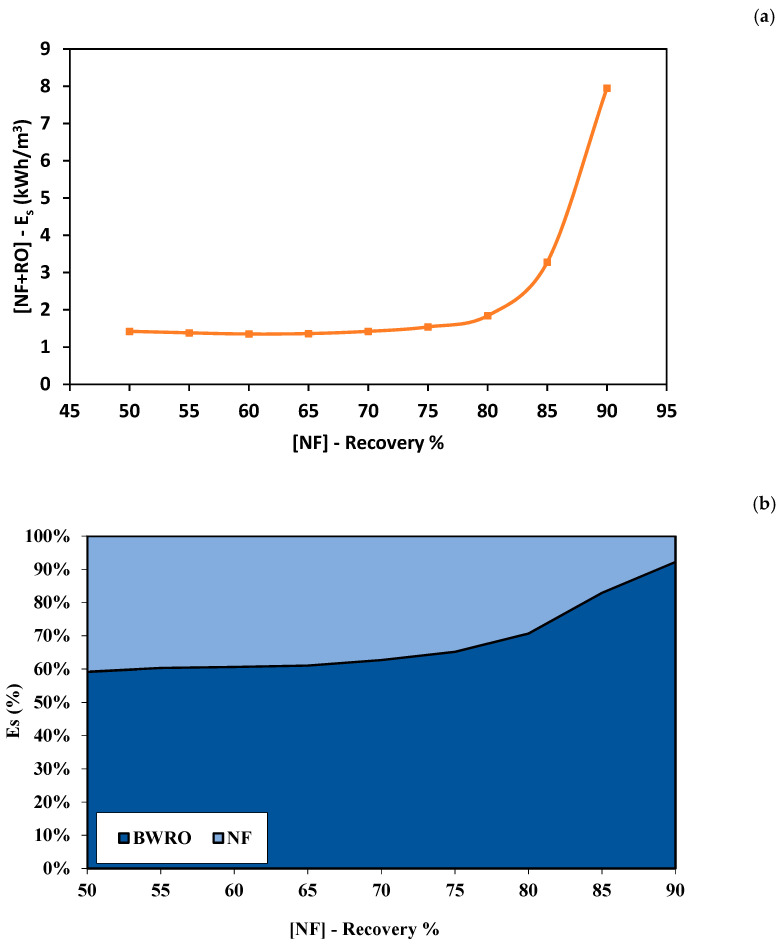
Energy consumption of the NF-RO hybrid process. (**a**) Total specific energy consumption of the hybrid process. (**b**) Percentage of the specific energy consumption.

Figure 5 shows the combined recovery rate of the hybrid NF-RO process using various recovery rates from the NF process. As the recovery rate of the NF process increased, the recovery rate of the hybrid process increased. At an NF recovery rate of 50%, the total recovery rate was 68.5%. As the NF recovery rate increased to 90%, the total recovery rate was 90%. This is due to the low recovery rate obtained using the RO process. The concentration of the mixed permeate was 178 ppm at an NF recovery rate of 50%. As the NF recovery rate increased to 60%, the concentration of the mixed permeate increased to 195 ppm. As the recovery rate of NF increased to 70%, the total concentration of permeate increased by 13.7%. The total concentration of permeate increased by 19% after increasing the NF recovery rate to 80%. At an NF recovery rate of 90%, the total concentration of permeate was 342 ppm, which is almost double the allowable limit recommended by FAO. It can be seen from Table 2 that the concentration of most ions was higher than the allowable limit, especially at a high recovery rate. As shown in Figure 6a, the total energy consumption of the hybrid process was between 1.42 kWh/m^3^ and 1.84 kWh/m^3^ for NF recovery rate between 50 and 80%. As the recovery rate of the NF process increased to 90%, the total energy consumption increased to 7.95 kWh/m^3^. Figure 6b shows the percentage of energy consumption for each process. Overall, the energy consumption percentage of RO was higher than NF. The percentage of energy consumption for RO was almost 61% between the NF recovery rate of 50% to 65%. The percentage of energy consumption for RO increased to 65% as the recovery rate of NF increased to 75%. Then the percentage of RO energy consumption increased significantly as the recovery rate increased beyond 80%. The percentage of RO energy consumption was 75% and 90% at a recovery rate of 80% and 90%, respectively. The total energy consumption increased at a higher recovery rate due to the high salinity of the brine used as a feed solution in the RO process. The energy consumption depends on the applied pressure that must overcome the natural osmotic pressure of the feed solution [46]. The osmotic pressure of the solution increases as the salinity of the solution increases. It can be concluded from the findings above that using RO for further extraction of permeate from the brine generated by the NF process is not a suitable solution. This is due to the high salinity of the final mixed permeate at the targeted recovery rate (i.e., 90%). The salinity of the mixed permeate was almost double the allowable limit that is recommended by FAO. In addition, the total energy consumption of the hybrid process was extremely high at the desired recovery rate.

#### 3.2.2. Hybrid Forward Osmosis (FO)–Reverse Osmosis (RO) process (Stage 2: Alternative 2)

**Figure 7:**
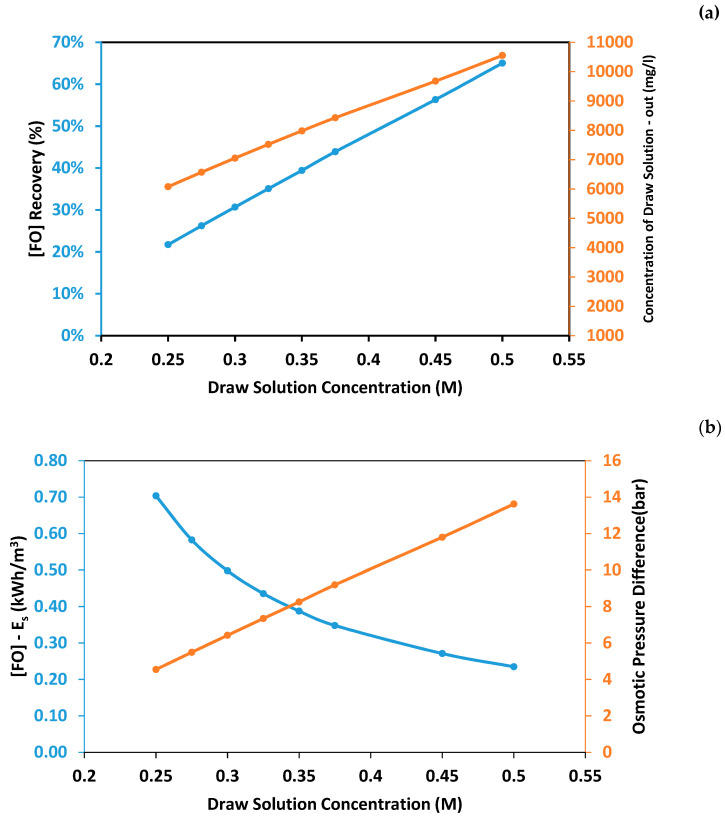

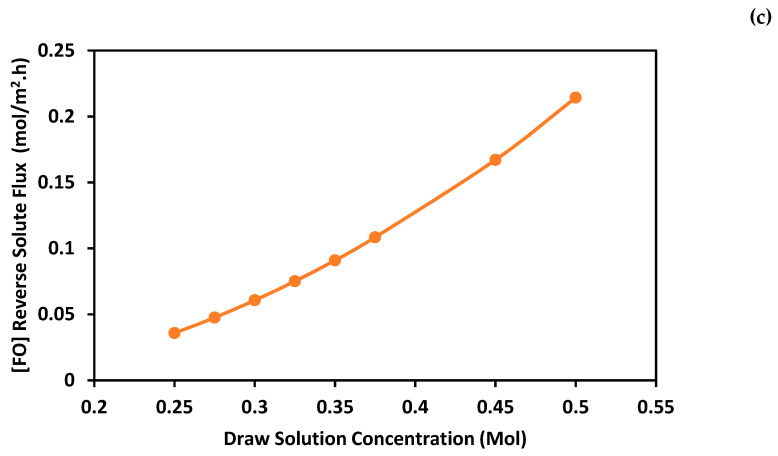
Performance of the hybrid forward osmosis–reverse osmosis (FO-RO) process using different feed solution concentrations. (**a**) Recovery rate and concentration of diluted draw solution; (**b**) Specific power consumption and osmotic pressure difference; (**c**) Reverse solute flux.

Forward osmosis was used to further extract pure water from the concentrated brine produced from the NF process before the use of the RO process. Forward osmosis was used in an attempt to maximize the overall recovery rate, enhance the final water quality, and reduce the energy consumption of the hybrid system. The concentrated brine generated by the NF process at a recovery rate of 75% was used as the feed solution in the FO process, while the draw solution was a synthetic NaCl solution with a concentration ranging between 0.25 M and 0.5 M. The results show that the FO recovery rate increased as the draw solution concertation increased (Figure 7a). This is due to a higher osmotic pressure gradient generated at high draw solution concertation [47]. The maximum recovery rate was 65% obtained using draw solution concentration 0.5 M, and the minimum recovery rate was 21.7% obtained using draw solution concentration 0.25 M. As observed from Figure 7b, the energy consumption of the FO process decreased as the draw solution concentration increased. This is due to the higher recovery rate obtained at higher draw solution concentration. The maximum energy consumption was 0.7 kWh/m^3^ obtained at a draw solution concentration of 0.25 M and decreased by 67.2% at a draw solution concentration of 0.5 M. As observed from Figure 7c, the reverse solute flux of the FO process increased as the draw solution concentration increased. This is due to higher water flux obtained at higher draw solution concentration. The minimum reverse solute flux was 0.0357 mol/m^2^.h obtained at a draw solution concentration of 0.25 M and increased by 6 folds at a draw solution concentration of 0.5 M. It is important to note that reverse solute flux will cause a loss in the osmotic driving force in the FO process. As shown in Figure 1, the regeneration of the draw solution using RO is a closed loop. Therefore, a frequent adjustment of the concentration of NaCl in the draw solution is required to maintain the water flux in the FO process.

**Figure 8:**
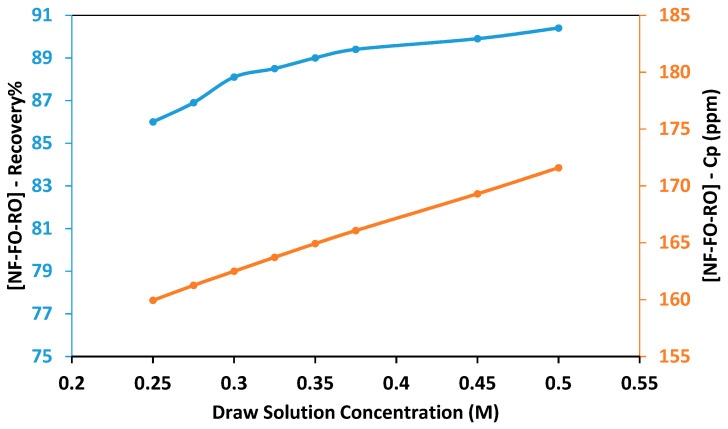
Total recovery rate and concentration of the mixed permeate solution obtained using hybrid NF-FO-RO process at different draw solution concentrations.

**Figure 9:**
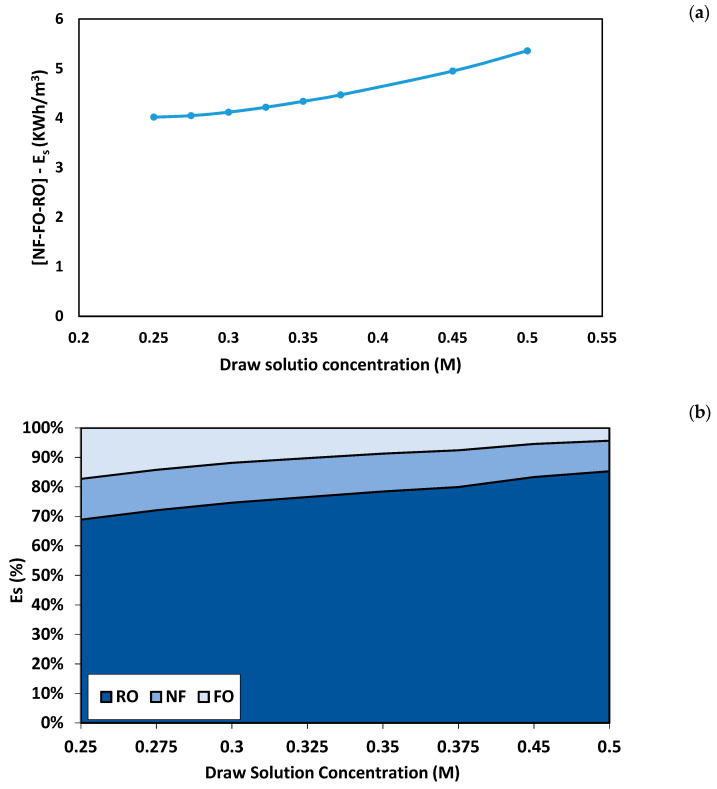
Energy consumption of the NF-FO-RO hybrid process. (**a**) Total specific energy consumption of the hybrid process. (**b**) Percentage of the specific energy consumption.

RO is the last stage of the hybrid NF-FO-RO process designed for the regeneration of the draw solution. The permeate from the RO process is mixed with the permeate from the NF process to produce the final product water. The running parameters of the RO process were designed so that the recovery rate of the FO process is equal to the recovery rate of the RO process. As shown in Figure 8, the total recovery rate of the hybrid system increased as the draw solution concentration increased from 0.25 M to 0.5 M. The minimum total recovery rate was 86% obtained using a draw solution concentration of 0.25 M, and the maximum total recovery rate was 90% obtained using draw solution concentration of 0.5 M. It can be seen from Table 3 that the concentration of all ions was within the allowable limits of irrigation water. Figure 9a shows the specific energy consumption of the hybrid NF-FO-RO process. The total energy consumption of the hybrid process was between 4.02 kWh/m^3^ and 4.34 kWh/m^3^ for a draw solution concentration between 0.25 and 0.35 M. At a draw solution concentration of 0.5 M, the total energy consumption was 5.36 kWh/m^3^. The specific energy consumption of seawater desalination using RO process is usually between 2.3 and 5.2 kWh/m^3^ for a recovery rate ranging between 45% and 60% [48]. The high energy consumption of the proposed hybrid system is mainly due to the high energy consumption in the RO process. Figure 9b shows the percentage of energy consumption for each process. Overall, the energy consumption percentage of RO was higher than NF and FO. The percentage of energy consumption for the NF process was constant because the recovery rate of the NF process was fixed at 75%. As the draw solution concentration increased, the percentage of energy consumption for the RO process increased, and the percentage of energy consumption for the FO process decreased. At a draw solution concentration of 0.25 M, the percentage of energy consumption for the RO process was 69%, and the percentage of energy consumption for the FO process was 18%. As the draw solution concentration increased to 0.5 M, the percentage of energy consumption for the RO increased to 82%, and the percentage of energy consumption for the FO process decreased to 4%.

## 4. Conclusions

In this paper, two hybrid processes were proposed for the supply of irrigation water from treated municipal wastewater. The hybrid process included two stages. In the first stage, the treated wastewater was treated using the NF process. In the second stage, more freshwater was reclaimed from the concentrated brine generated by the NF process using two different alternatives. The first alternative included an RO process, while the second alternative included a hybrid FO-RO process. It was found that the first alternative was not suitable for the further extraction of permeate from the brine generated by the NF process. This was due to the high salinity of the final permeate at the targeted recovery rate of 90%. The salinity of the final permeate was almost double the allowable limit that is recommended by FAO. In addition, the total specific energy consumption (*Es*) of the NF-RO hybrid process was high, with a value of 7.95 kWh/m^3^ at the targeted 90% recovery rate. Using the NF-FO-RO hybrid system reduced the specific energy consumption by 27%, with a value of 5.36 kWh/m^3^ at the same 90% recovery rate. In addition, the quality of the final permeate using the NF-FO-RO hybrid system was within the FAO standards. The findings mentioned earlier were obtained using 0.5 M NaCl as a draw solution concentration. This was used as a draw solution due to the low energy consumption and water quality suitable for irrigation purposes. In future studies, it is recommended to test another type of RO membrane for the regeneration of the draw solution. The utilization of a brackish water RO membrane could decrease the energy consumption of the RO process; however, the final permeate water quality must be evaluated due to the lower rejection rate of salts. In addition, the energy consumption of the RO process can be reduced by using an energy recovery device. The performance of the proposed hybrid process must be evaluated using benchtop then pilot-scale setups to further evaluate the effect of membrane fouling, rejection rate for different ions, and concentration polarization phenomena.

## Figures and Tables

**Table 1:** Characteristics of tertiary treated sewage effluent (Feed Water).

Parameter	Value	Max Limit [38,39,40]	Standard Method
**TDS (ppm)**	1461 ± 5	175	APHA 2540 C. Total Dissolved Solids Dried at 180 °C
**Turbidity (NTU)**	0.2 ± 0.1	2	APHA 2130 B. Nephelometric Method
**EC (mS/cm)**	2.56 ± 0.2	0.7	APHA 2510 B. Conductivity
**Fluoride (ppm)**	0.27 ± 0.2	1.5	APHA 4110 Determination of anions by ion chromatography
**Chloride (ppm)**	897.5 ± 0.2	102	
**Bromide (ppm)**	0.96 ± 0.2	1	
**Nitrate (ppm)**	25.84 ± 0.2	20	
**Sulfate (ppm)**	320.3 ± 0.2	20	
**Sodium (ppm)**	200.3 ± 0.2	65	APHA 3120 Determination of metals by plasma emission spectroscopy
**Potassium (ppm)**	12.4 ± 0.2	10	
**Calcium (ppm)**	87.7 ± 0.2	40	
**Magnesium (ppm)**	21.4 ± 0.2	24	

**Table 2:** Concentration of various ions and cations in the final mixed permeate obtained using hybrid nanofiltration–reverse osmosis (NF-RO) Process at Different Recovery %.

Recovery% (NF)	Recovery% (RO)	Overall Recovery%	Concentration of Ions in the Permeate Water (ppm)							
			F^−^	K^+^	Mg^2+^	Ca^2+^	SO_4_^2−^	Na^+^	Cl^−^	TDS
50	37.1	68.5	0.1	3.8	1.4	5.6	10.6	43.2	56	156.1
55	38.2	72.2	0.1	4.8	1.7	7	13.3	53.7	69.8	194.1
60	38.5	75.4	0.1	6.1	2.2	9	17.3	69.1	90.1	249.7
65	37.6	78.2	0.1	8.2	3.1	12.3	23.6	93.4	122	337.5
70	35.3	80.6	0.2	11.7	4.4	17.9	34.2	133.3	175	482.1
75	31	82.8	0.2	17.8	7	28.4	54.6	205.6	271.8	746
80	24.1	84.8	0.4	32.4	13.3	53.9	104	378.7	505.5	1376
85	11.5	86.7	0.9	70.2	32.2	131.3	257	852.6	1160	3124
90	4.27	90.4	2.2	180.1	111.2	453.6	925.6	2400	3381	9057
**Limits [38,39,40]**			1.5	10	24	40	20	65	102	175

**Table 3:** Concentration of various ions and cations in the final mixed permeate obtained using the hybrid nanofiltration–forward osmosis–reverse osmosis (NF-FO-RO) process at an NF recovery rate of 75% and different draw solution concentrations.

Draw Solution Concentration (M)	Overall Recovery%	Recovery% (NF)	Recovery% (FO)	Recovery% (RO)	Concentration of Ions in the Permeate Water (ppm)							
					F^−^	K^+^	Mg^2+^	Ca^2+^	SO_4_^2−^	Na^+^	Cl^−^	TDS
0.25	86.0	75	21.7	21.7	0.038	2.95	0.92	3.61	5.87	51.6	76.8	159.9
0.275	86.9	75	26.2	26.2	0.038	2.97	0.92	3.63	5.91	52.0	77.5	161.3
0.3	88.1	75	30.7	30.7	0.039	2.99	0.93	3.65	5.94	52.4	78.1	162.5
0.325	88.5	75	35.1	35.1	0.039	3.00	0.93	3.67	5.97	52.9	78.8	163.7
0.35	89.0	75	39.4	39.4	0.039	3.02	0.94	3.69	6.00	53.3	79.4	164.9
0.375	89.4	75	43.9	43.9	0.039	3.03	0.94	3.71	6.03	53.7	80.0	166.1
0.45	89.9	75	56.3	56.3	0.040	3.07	0.95	3.76	6.12	54.8	81.7	169.3
0.50	90.4	75	65.0	65.0	0.040	3.10	0.96	3.79	6.18	55.6	82.8	171.6
**Limits [38,39,40]**					1.5	10	24	40	20	65	102	175

## Data Availability

All data generated or analyzed during this study are included in this published article.
